# Heterotopic pregnancy in natural conception

**DOI:** 10.4103/0974-1208.39595

**Published:** 2008

**Authors:** Govindarajan MJ, Rajan R

**Affiliations:** No. 6, Sreenivasa Nilaya, 3rd cross, Bima Jyothi, LIC Colony, Basaveswar Nagar, Bangalore, India

**Keywords:** Adnexal mass, assisted conception, heterotopic

## Abstract

Heterotopic gestation, although common with assisted reproductive techniques, is very rare in natural conception. A high index of suspicion can help in timely diagnosis and appropriate intervention. We report a case of heterotopic pregnancy in a 22-year-old woman presented with hemoperitoneum from ruptured tubal pregnancy with live intrauterine gestation at 10 weeks of amenorrhea, diagnosed on ultrasound examination.

Heterotopic pregnancy is defined as the coexistence of intrauterine and extrauterine gestation. The incidence of heterotopic pregnancy is very low. The frequency was originally estimated on theoretical basis to be 1 in 30,000 pregnancies. We present a rare case of heterotopic pregnancy with live intrauterine gestation and ruptured left adnexal gestation in a natural conception.

## CASE REPORT

**Figure 1:**
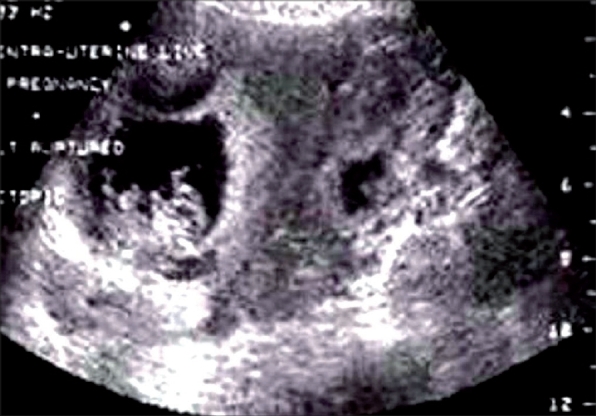
TVS showing an intrauterine gestation and an adnexal mass

**Figure 2:**
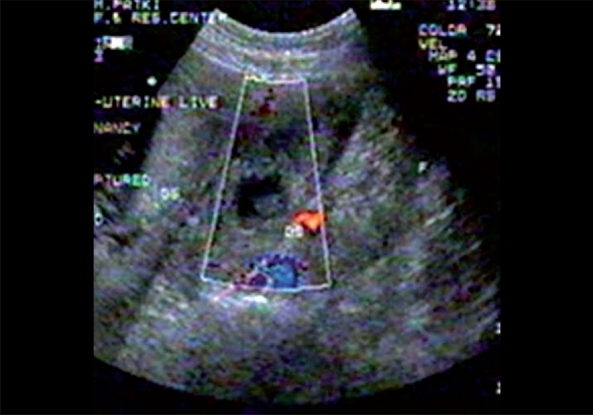
TVS color Doppler showing adnexal mass with color flow in the trophoblastic tissue

A 22-year-old woman with 10 weeks of amenorrhea presented for emergency ultrasound scan of pelvis with clinical features of shock. Urine pregnancy test was positive. Transabdominal ultrasound revealed moderate amount of free fluid in the peritoneal cavity with a live intrauterine gestation of about 10 weeks. A complex left adnexal mass was also noted. The transvaginal ultrasound confirmed the findings [Figure 1]. The Doppler study of left adnexal mass showed low resistance flow [Figure 2]. Provisional diagnosis of a heterotopic pregnancy with ruptured left ectopic gestation was suggested in view of clinical history, moderate amount of free intraperitoneal fluid, and an intrauterine gestation. The patient underwent emergency laparoscopy. There was ruptured left-sided tubal pregnancy with hemoperitoneum and laparoscopic tubal surgery was performed; the intrauterine live gestation was allowed to continue. The patient delivered a healthy live baby at term.

## DISCUSSION

A heterotopic gestation is difficult to diagnose clinically. Typically, laparotomy is performed because of tubal pregnancy. At the same time, uterus is congested, softened, and enlarged; ultrasound examination can nearly always show gestational products in uterus.

The incidence was originally estimated on theoretical basis to be 1 in 30,000 pregnancies. However, more recent data indicate that the rate is higher due to assisted reproduction and is approximately 1 in 7000 overall and as high as 1 in 900 with ovulation induction.[1,2]

The increased incidence of multiple pregnancy with ovulation induction and IVF increases the risk of both ectopic and heterotopic gestation. The hydrostatic forces generated during embryo transfer may also contribute to the increased risk.[1]

There may be an increased risk in patients with previous tubal surgeries.[3]

Heterotopic pregnancy can have various presentations. It should be considered more likely (a) after assisted reproduction techniques, (b) with persistent or rising chorionic gonadotropin levels after dilatation and curettage for an induced/spontaneous abortion, (c) when the uterine fundus is larger than for menstrual dates, (d) when more than one corpus luteum is present in a natural conception, and (e) when vaginal bleeding is absent in the presence of sings and symptoms of ectopic gestation.[4]

A heterotopic gestation can also present as hematometra and lower quadrant pain in early pregnancy.[5]

Most commonly, the location of ectopic gestation in a heterotopic pregnancy is the fallopian tube. However, cervical and ovarian heterotopic pregnancies have also been reported.[6,7]

Majority of the reported heterotopic pregnancies are of singleton intrauterine pregnancies. Triplet and quadruplet heterotopic pregnancies have also been reported, though extremely rare.[8,9] It can be multiple as well.[4] They can be seen frequently with assisted conceptions.

Intrauterine gestation with hemorrhagic corpus luteum can simulate heterotopic/ectopic gestation both clinically and on ultrasound.[10] Other surgical conditions of acute abdomen can also simulate heterotopic gestation clinically and hence the difficulty in clinical diagnosis. Bicornuate uterus with gestation in both cavities may also simulate a heterotopic pregnancy.

High resolution transvaginal ultrasound with color Doppler will be helpful as the trophoblastic tissue in the adnexa in a case of heterotopic pregnancy shows increased flow with significantly reduced resistance index.[2]

The treatment of a heterotopic pregnancy is laparoscopy/laparotomy for the tubal pregnancy.[4]

The illustrated case did not have any risk factor for the heterotopic gestation and presented with ruptured tubal pregnancy with hemodynamic instability due to hemoperitoneum.

A heterotopic pregnancy, though extremely rare, can still result from a natural conception; it requires a high index of suspicious for early and timely diagnosis; a timely intervention can result in a successful outcome of the intrauterine fetus.[11]
